# Mammary tumor associated *Hspb1* mutation and screening of eight cat populations of the world

**Published:** 2016

**Authors:** R. Saif, A. R. Awan, L. Lyons, B. Gandolfi, M. Tayyab, M. Ellahi Babar, M. Wasim

**Affiliations:** 1 Department of Biotechnology, Virtual University of Pakistan, Lahore, Pakistan;; 2 Institute of Biochemistry and Biotechnology, University of Veterinary and Animal Sciences, Lahore, Pakistan;; 3 Department of Veterinary Medicine & Surgery, College of Veterinary Medicine, University of Missouri-Columbia, Columbia, MO 65211, USA

**Keywords:** Cat mammary lesions, Cat population, *Felis catus*, *Hsp27* mutation, *Hspb1* screening

## Abstract

Current research highlights the *Hspb1* based screening of eight cat populations of the world to investigate the association of newly found locus within cat mammary tumors. Total 180 cats were screened on the basis of *Hspb1* 4 bp deletion locus (1514-1517del4) which was observed in six mammary tumor cases in Siamese cat breed. Case-control association study revealed the non-significance with P=0.201 and an overall mutant allele frequency of 0.30 ranging from 0.20-0.40 was observed in other cat populations. Similarly, HWE was also obeyed in combined population samples with P=0.860 and found non-significant with range of 0.429-0.708 in other non-Pakistani cat populations as well. These results might be helpful to understand the association of this novel locus in a better way with large sample size of cases and may also serve as a potential marker for mammary tumor diagnosis, particularly in cats and generally in all other animal populations in comparative genetics and genomics context.

## Introduction

The word “cancer” is so fearsome and attention seeking for victims, practitioners and guardians, regardless of the type of species involved. Mammary adenocarcinoma is the third most common cancer in domestic cats (Bell, 2008 ▶), out of total incidences, 80% are malignant while 10-20% appeared as benign cases which ultimately turned into malignant form (Giménez et al., 2010 ▶). Malignant tumors are equally lethal in animals as in humans and some animal cancer like mammary gland tumor in cat is a rationale model to study human cancer because of its resemblance in cell morphology, histopathology, risk factors, prognosis, invasion and response to chemotherapy and other adopted treatment modalities (Burrai et al., 2010 ▶; Shafiee et al., 2013 ▶). Breast cancer is a significant health concern in humans and small animals, especially in Siamese cat, so special emphasis was given to ascertain cancer associated sequence variants of heat shock protein-beta1 (*Hspb1*) gene in this neoplasm (Rutteman and Misdorp, 1992 ▶; Zappulli et al., 2005 ▶). Mammary tumor has different aetiology, some toxic chemical and physical factors may involve, hormonal imbalances in the older stages of life and radiation exposure to stray cats is considered a momentous factor in its outbreak (Rutteman and Misdorp, 1992 ▶).

Genetic causes may also play a significant role in cancer proliferation, in which an affected gene copy is transmitted through generations as explained by Knudson multi-hit hypothesis (Knudson, 1971 ▶). Clinical-trial based treatment modalities are well documented for mammary adenocarcinoma but genetic and diagnostic biomarkers are infrequently used in molecular oncology (Hughes and Dobson, 2012 ▶). So, it is one of the major researches emphasized in the current era to diagnose cancer through molecular diagnostics biomarkers methodologies.

The Siamese breed is comparatively more prone to this ailment due to unidentified reasons. In general, environmental toxins, second-hand smoke, excessive grooming and surface licking might be putative reasons of tumor growth in animals (Hughes and Dobson, 2012 ▶). This breed is very peculiar in its small face, light brown tail and paws, short haired lean body, slightly flat erected pinna, triangular shaped head and face with a thin pointed chin. The higher tendency of mammary gland tumor in this breed attracted the attention of veterinary cancer genomics experts to seek its putative causes, whether it has a familial or sporadic origin. So, *Hspb1* gene characterization and screening effort were conducted in this study which may not only be helpful in this particular breed but informative in other animals and even in human counterpart.

Among all stress proteins, *Hspb1* gene is characterized here, which is located on chromosome E3 at 973,860-975,895 position, having only one transcript of 1411 bp with 3 exons which cipher 205 amino acids, having 88% and 86% sequence identity with dog and human counterpart, respectively (Fernández and Birney, 2010 ▶). This protein plays its significant role in many processes of tumor development, especially in the cell cycle regulation, immunosurveillance, cell differentiation and in apoptotic pathways. High level of this protein is reported in regression stage of cancer and is linked with anti-apoptotic activities as well (Garrido et al., 2003 ▶).

Novel tumor-associated mutations were found and screened in the eight cat populations of the world including Pakistani inbred and random cohort to have a clear picture of the genomic structure of *Hspb1* gene in *Felis catus*. Screening tests are often used in clinical practices to assess the likelihood, whether a patient has a particular medical condition or not. The rationale behind that, if the disease is identified early before the manifestation of symptoms, the adopted treatment may lead to cure or improve the quality of the patient’s life.

## Materials and Methods

**Sample collection (cancer case vs. control)**

Six Siamese cats were presented from different owners at Pet Centre, University of Veterinary and Animal Sciences (UVAS), Lahore, Pakistan with the symptoms of manifesting, firm and nodular lump in the breast tissue, overlying skin, red swollen nipples, yellowish fluid secretion with other particular signs and symptoms of bowel/bladder changing habits, difficult breathing, sniffing and chronic weight loss. Tumorous mass was collected through standard protocol of sample collection after surgical resections. Then all tissue masses were stored at -86°C for down-steam DNA extraction (Sambrook and Russell David, 1989 ▶).

**DNA extraction**

TaiGen genomic DNA tissue kit (TaiGen Biotechnology Co., Ltd., Neihu Dist., Taipei, Taiwan) was used to extract the DNA from the core tumorous tissues according to manufacturer guidelines (Vogelstein and Gillespie, 1979 ▶). While genomic DNA from blood was also extracted using GF-1 tissue blood combi DNA extraction kit (V*i*vant*i*s Technologies SDN, BHD, Selangor DarulEhsan, Malaysia). DNA concentration was measured and confirmed by NanoDrop spectro-photometer (Thermo Scientific, Wilmington, DE, USA) and agarose gel electrophoresis methods, respectively.

**Primer designing**

Long-range primer set of 2303 bp product size was designed from the DNA sequence ID ENSFCAT00000026034 of *Hspb1* gene taken from ENSEMBLE genome browser (Fernández and Birney, 2010 ▶) using Primer3 and NetPrimer software (PREMIER Biosoft International, Palo Atlo, CA) (Rozen and Skaletsky, 2011 ▶).

Additionally, a set of labeled primer was also designed for a required fragment length analysis for screening the population. The total amplicon length was 232 bp, which captured newly found 4 bp deletion locus in *Hspb1*. This primer pair amplified the specific region of interest and results were analyzed using “STRand” software (STRand Nucleic AcidAnalysis Software, University of California, Davis (http://www.vgl.ucdavis. edu/STRand).

**PCR amplification, protocol and reagent con-centrations**

Long-range polymerase chain reaction (PCR) was conducted using Applied Biosystem thermocycler at 94°C temperature for 2 min as initial denaturation, then 10 cycles of 94°C as cyclic denaturation for 10 s, annealing at 61°C for 30 s and extension temperature at 68°C was adopted for 3 min keeping in mind the product size of 2303 bp. Then 30 cycles were run in continuity at 94°C as cyclic denaturation for 10 s, annealing at 59°C for 30 s and extension temperatures at 68°C for 3 min with an increment of 20 s extension time per cycle, at the end final extension was given at 72°C for 5 min and the reaction was held at 4°C. Optimized long-range PCR kit with dNTPs was used, which contained high-fidelity long-range polymerase of 5 U/μL with final con-centration of 1.8 U, PCR 10X enhancer-A with final concentration of 1X, PCR additive dimethyl sulfoxide (DMSO) for GC-rich region amplification and 10X reaction buffer with final concentration of 1X were used to amplify the *Hspb1* gene (Lee et al., 1990 ▶; Ozcelik et al., 2012 ▶).

**Gel electrophoresis, PCR amplicon sequencing and data analysis**

**Fig. 1:**
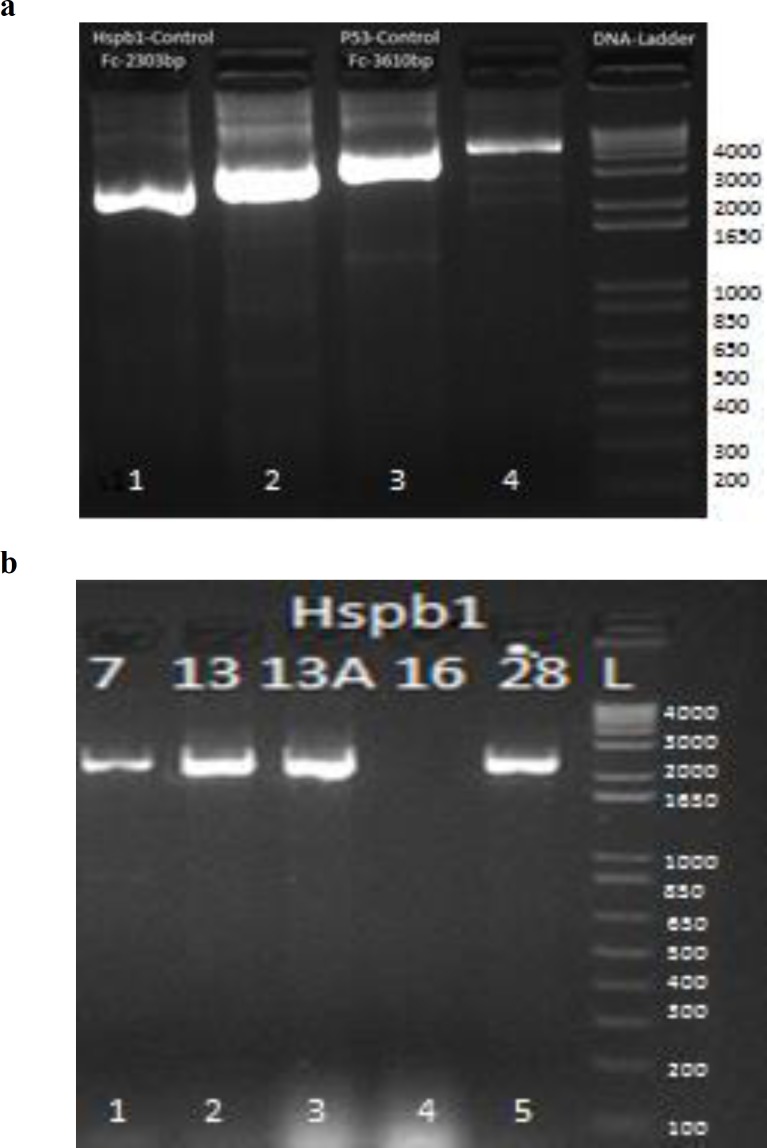
Gel electrophoresis of *Hspb1* gene, (a) Long-range PCR amplified product of *Hspb1* gene in mammary tumor samples and control with 1 Kb Gene Ruler DNA ladder. Well # 1 has a product size of 2303 bp of *Hspb1* gene in normal control, (b) specific product of 2303 bp of *Hspb1* gene in tumor samples are shown in well # 1, 2, 3, 5

Electrophoresis of PCR product was conducted in 1.5% agarose gel for 50 min at 80 V (Fig. 1a). After obtaining the specific product, product was purified for sequencing by treating with ExoSAP (Affymetrix, Santa Clara, CA, USA) reagent (Bell, 2008 ▶). Sanger sequencing was done with ABI BigDye terminator sequencing kit (Applied Biosystems, Foster City, CA, USA). Sequence analysis was performed using “Sequencher” 5.1 software (Gene Codes Corporation, Ann Arbor, MI, USA) to ascertain the polymorphism and its other statistical attributes (Sambrook and Russell David, 1989 ▶; Lee et al., 1990 ▶).

**Amplification for fragment analysis and gel electrophoresis**

**Fig. 2:**
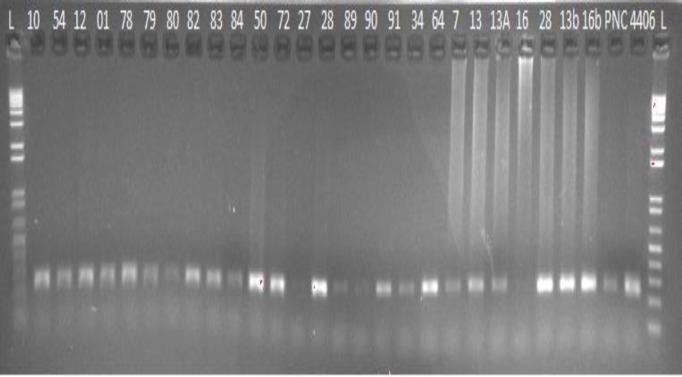
Amplification of 232 bp fragment of 4 bp deletion in *Hspb1* gene in *Felis catus* through labeled primers

Fluorescently labeled primer was designed to genotype the GTCT 4 bp deletion mutation in cat *Hspb1* gene by keeping in mind, that the amplicon length should not be greater than 250 bp. Eventually, a set of labeled primer was designed using NetPrimer software, which amplified 232 bp fragments. Standard end-point PCR was conducted to amplify the required fragment for cat population screening and finally, these amplicons were run in 1.5% agarose gel at 80 V (Fig. 2).

## Results

**Detection of** ***Hspb1*** **sequence variants**

*Hspb1* gene was amplified through PCR with the help of specific primers and all cancerous samples were sequenced in which a 4 bp deletion locus in intron 2 of *Hspb1* gene was observed (Figs. 1a and b).

**Screening of eight cat populations of the world**

Two hundred and eight normal cat samples from Pakistan, France, Philippines, Brunei, Iraq, Oman, South Korea and USA were screened with labeled primers, which were designed with amplicon length of 232 bp and genotyped with ABI 3730 XL genetic analyzer (Fig. 2).

**Fig. 3:**
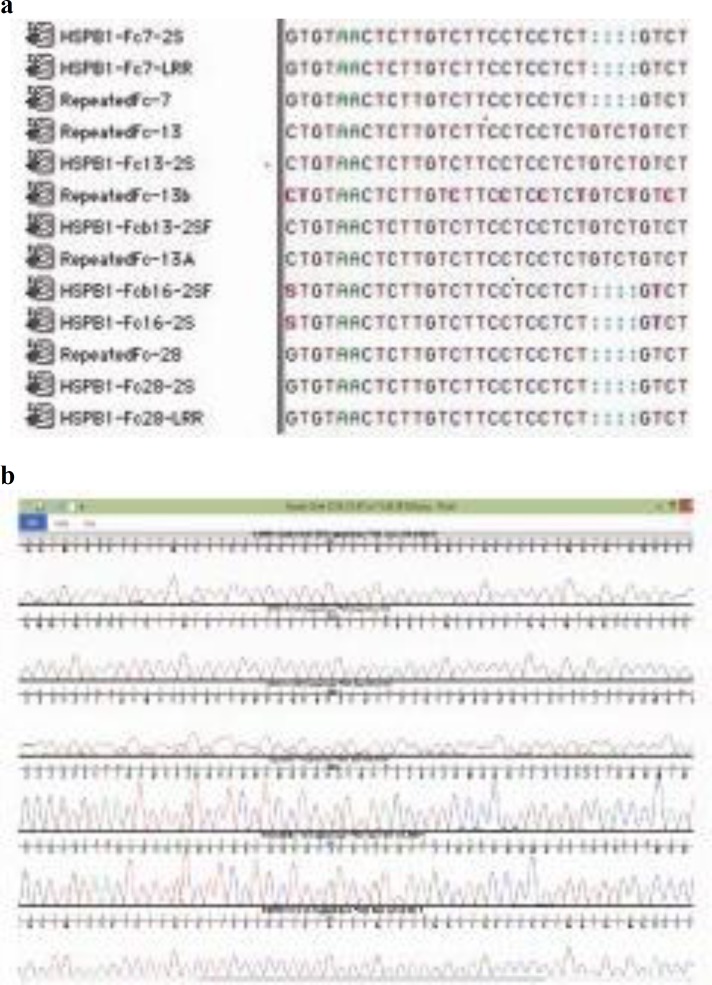
Sequence alignment through “Sequencher” software (a) Snapshot of multiple sequence alignment of 1415-1417 4 bp deletion locus and (b) chromatograms of the same locus in *Hspb1* gene in *Felis catus*

Which were further reconfirmed by designing a new set of primers from this variant locus of 4 bp deletion and same results were obtained which include two tumorous tissues samples (CP13, CP13A), that were found wild-type without 4 bp deletion at 1514-1517 loci in intron 2. Two tumorous tissue samples (CP16, CP16b) were found heterozygous at this locus (one allele wild-type, second allele with 4 bp deletion), while the remaining two tumorous tissues samples (CP7, CP28) were observed homozygous mutant with 4 bp deletion (Figs. 3a and b).

**Fig. 4:**
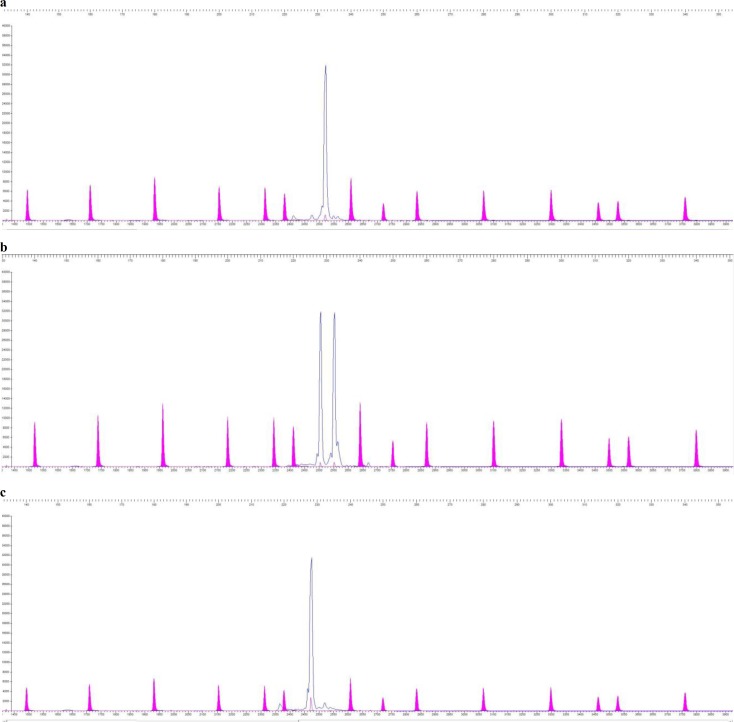
“(a) STRand” software showing a single peak of wild-type homozygous fragment of 232 bp in blue, (b) heterozygous fragment of 232 bp and 228 bp, (c) homozygous mutant fragment of 228 bp in *Felis catus*, while varying size standards are shown in purple in (a, b and c

Total of seventy-nine, sixteen and eighty-two individuals were found wild-type, homozygous mutant and heterozygous respectively, while thirty-one sample peaks were not readable through “STRand” software. Fragment peaks are shown in (Figs. 4a, b and c), which depict homozygous (wild-type), heterozygous and homozygous mutants respectively. Cat population data was analysed and shown in (Table 1).

**Case-control association**

Six cancerous cats harboured mutant allele frequency of 0.50 and appeared as not-significant in Pakistani population with a P=0.333 which depicts that subject locus may not be predominantly associated with mammary tumor cases in cats. Similarly, cancerous population also obeyed Hardy-Weinberg Equilibrium (HWE) with a P=0.414. Cancer group association was calculated through Fisher’s Exact test which showed not-significant with an overall P=0.201 and accumulative mutant allele frequency of 0.30 in all populations. Pakistani (P=0.333), French (P=0.071), Philippine (P=0.740), Brunei (P=0.163), Iraqi (P=0.543), Omani (P=0.119), and USA (P=0.163) cat populations showed not-significant results of subject locus with mammary tumor while South Korean cat population showed significant results with P=0.042.

**Table 1:** Association of *Hspb1* 4 bp deletion locus in different cat populations of the world

Population cohorts	Total number of samples	Wild type	Homo-mutant	Hetero	Failed peaks	Mutant allele frequency	Fisher’s Exact (P-value) (Pak-MT vs. other populations-N)	Fisher’s Exact (P-value) (Pak-N vs. other populations-N)	HWE (P-value at 1df)
Mammary tumor cases	06	2	2	2	--	0.50	--	--	0.414 (NS)
Pakistan	63	20	4	22	17	0.33	0.333 (NS)	--	0.549 (NS)
France	24	15	3	3	3	0.21	0.071 (NS)	0.222 (NS)	0.008 (S)
Philippines	22	4	0	16	2	0.40	0.740 (NS)	0.432 (NS)	0.003 (S)
Brunei	32	18	4	7	3	0.26	0.163 (NS)	0.465 (NS)	0.046 (S)
Iraq	32	10	4	17	1	0.40	0.543 (NS)	0.391 (NS)	0.437 (NS)
Oman	12	6	0	4	2	0.20	0.119 (NS)	0.772 (NS)	0.429 (NS)
South Korea	4	4	0	0	0	0	0.042 (S)	0.101 (NS)	-(NS)
USA	19	10	1	8	0	0.26	0.163 (NS)	1.000 (NS)	0.708 (NS)
Total	208	87	16	77	28	0.30	0.201 (NS)	0.704 (NS)	0.860 (NS)

S: Significant, NS: Not-significant, MT: Mammary tumor, and N: Normal. http://www.socscistatistics.com/tests/fisher/Default2.aspx

**Mutant allelic frequency distribution**

Total 186 (180-normal and 6-cases) cats were including in the calculation comprised of 46, 21, 20, 29, 31, 10, 4, and 19 from Pakistani, French, Philippine, Brunei, Iraqi, Omani, South Korean and American cats respectively. The overall mutant allele frequency of 0.30 was observed in all populations. Philippine and Iraqi population harbour maximum mutant allele frequency of 0.40 in each. Similarly, Pakistani cat population has frequency of 0.33 followed by 0.26 in American and Brunei cats, then 0.21, 0.20 in French and Omani population, respectively (Table 1).

**Comparison among eight cat populations**

Association between the Pakistani cats with other populations was carried out through Fisher’s Exact test. Pakistani normal cat population appeared not-significant with P=0.222 when compared with French population which depicts that these two populations have the same population dynamics. Similarly, when Pakistani population association was measured with Philippine, Brunei, Iraq, Oman, South Korea and American, all appeared as not-significant with P=0.432, 0.465, 0.391, 0.772, 0.101, 1.000, respectively. An overall P=0.704 was observed in Pakistani and other samples from all populations which clearly inferred from this association that 46 Pakistani cat samples randomly bred unrelated animals.

**Hardy-Weinberg equilibrium**

Randomly selected unrelated samples were collected from all populations which were also reflected by Fisher’s Exact Chi-square P-values. All populations showed not-significant results with an overall P=0.860. Hardy-Weinberg equilibrium also showed not-significant result with P=0.414, 0.549, 0.437, 0.429, and 0.708 in Pakistani tumor cases, Pakistani normal, Iraqi, Omani, South Korean, and US population, respectively. Which means that these populations are randomly bred and totally unrelated animals, the sample size was satisfactory, no inbreeding, no stratification, subject locus is not under selection and no migrations were observed in these populations. While three populations of French, Philippines and Brunei showed deviations from the HWE with P=0.008, 0.003 and 0.046 respectively.

## Discussion

Labeled primers were used to screen the 1514-1517 4bp deletion loci in *Hspb1* intron 2 found in cat mammary adenocarcinoma. This gene has well renowned tumorigenic, anti-apoptotic functions and a potential marker for screening of cat populations in this particular ailment. Mutation may damage gene structure and functions of proto-oncogene and turns them to an oncogene one way or the other. This process go-on all the time, but usually remained stopped in fully functional proto-oncogenes. On the other hand, mutations alter the tumor suppressing function, and genes lose their function of cell growth breaks, which usually direct the cells to stop growing due to their ended life span.

The current research endeavour enlighten us that 4 bp deletion in intron 2 at splice site of *Hspb1* gene might be associated with mammary tumor in *Felis catus*. Normally, sensitivity and specificity are two commonly used measures to evaluate the performance of screening tests. The sensitivity reflects the probability that the screening test will be positive among diseased, while specificity reflects the probability that the screening test will be negative among those who do not have the disease (Zauber et al., 2008 ▶; Corporation, 2010 ▶). In this study, a total of 208 individuals were screened, out of which, 180 samples were included in allelic and genotypic population association calculations. Seven other populations were selected by putative relationship with Pakistani cats, especially the French population due to the hundred years of France and Great Britain ruling over this territory of the Indian subcontinent, while Oman and Iraq are neighbouring countries. The American, Brunei and South Korean populations were selected as an out-group source. Results revealed that Omani cats have the lowest allele frequency of 20% for this mutant allele, while Iraqi and Philippine population have the maximum of 40%. All cats included in screening belongs to different breeds, but 19 US cats were solely Siamese breed, this population has 26% of mutant allele and appeared as not-significant as compared to Pakistani tumorous cat population.

Almost one-third (32%) of the screened cats carried this mutant allele, which showed strongly not-significant results (P=0.201) associated with mammary tumor through this novel locus. While only one population of South Korea showed significant association which might be not so informative due to smaller population size. One of the worth mentioning points is that, no doubt, the French population showed not-significant association, but this association is relatively very weak (P=0.071) as compared to other highly strong not-significant association.

This locus of 4 bp deletion in intron 2 of *Hspb1* gene showed not-significant association with mammary tumor in cats. Overall, 32% mutant allele frequency was observed in all populations, but South Korean cats showed significant association, it might be less informative due to small sample size. Five cat populations, Pakistani, Iraqi, Omani South Korean and American obeyed the HWE while three populations of France, Philippines and Brunei showed deviation, that might be due to excessive inbreeding, population migration, stratification and selection pressure or small sample size.
